# Computational Exposure Science: An Emerging Discipline to Support 21st-Century Risk Assessment

**DOI:** 10.1289/ehp.1509748

**Published:** 2015-11-06

**Authors:** Peter P. Egeghy, Linda S. Sheldon, Kristin K. Isaacs, Halûk Özkaynak, Michael-Rock Goldsmith, John F. Wambaugh, Richard S. Judson, Timothy J. Buckley

**Affiliations:** Office of Research and Development, U.S. Environmental Protection Agency, Research Triangle Park, North Carolina, USA

## Abstract

**Background:**

Computational exposure science represents a frontier of environmental science that is emerging and quickly evolving.

**Objectives:**

In this commentary, we define this burgeoning discipline, describe a framework for implementation, and review some key ongoing research elements that are advancing the science with respect to exposure to chemicals in consumer products.

**Discussion:**

The fundamental elements of computational exposure science include the development of reliable, computationally efficient predictive exposure models; the identification, acquisition, and application of data to support and evaluate these models; and generation of improved methods for extrapolating across chemicals. We describe our efforts in each of these areas and provide examples that demonstrate both progress and potential.

**Conclusions:**

Computational exposure science, linked with comparable efforts in toxicology, is ushering in a new era of risk assessment that greatly expands our ability to evaluate chemical safety and sustainability and to protect public health.

**Citation:**

Egeghy PP, Sheldon LS, Isaacs KK, Özkaynak H, Goldsmith M-R, Wambaugh JF, Judson RS, Buckley TJ. 2016. Computational exposure science: an emerging discipline to support 21st-century risk assessment. Environ Health Perspect 124:697–702; http://dx.doi.org/10.1289/ehp.1509748

## Introduction

Traditional, hazard-driven, single-chemical risk assessment practices that follow the 1983 National Research Council (NRC) paradigm (NRC 1983) cannot keep pace with the vast and growing numbers of chemicals in commerce (Harvey et al. 1995; NRC 1984). A well-defined, quantitative, and defensible means of identifying chemicals with the greatest risk potential is needed (Judson et al. 2009; NRC 2007b, 2009; van Leeuwen et al. 2007), with exposure considerations providing a critical context for allocation of limited resources (Cohen Hubal 2009; Dellarco et al. 2010; Egeghy et al. 2011; Sheldon and Cohen Hubal 2009). However, elevating the role of exposure science will require the development and application of efficient and reliable computational models that make full use of the rich and growing sources of accessible exposure-relevant information (NRC 2012). We propose a new discipline, called “computational exposure science,” that expands the knowledge and current methods used in the field of exposure assessment by bringing in novel data sources and new computational technologies.

A key driver of computational exposure science is the reinvigoration of interest in the significant role played by the environment in disease etiology that has accompanied the conceptualization of the “exposome” (Prüss-Üstün and Corvalán 2006; Rappaport and Smith 2010; Wild 2005). The exposome refers to the totality of an individual’s environmental exposures from conception onwards. Integrating external agents, internal response, and the social, cultural, and ecological contexts of exposure, it was conceived to complement the genome for investigation of disease etiology (Wild 2012). The rise in increasingly prevalent diseases, such as autism, asthma, and childhood leukemia (Hertz-Picciotto and Delwiche 2009; Meeker 2012; Perrin et al. 2007), against a backdrop of widespread human exposure to industrial chemicals, as revealed by biomonitoring surveys (Becker et al. 2007; Centers for Disease Control and Prevention 2009; Park et al. 2012), confers a societal obligation to comprehensively understand exposures. Traditional strategies for evaluating chemical exposures have not provided even the most basic information about exposures for the vast majority of chemicals in commerce (Egeghy et al. 2012; Muir and Howard 2006; Schwarzman and Wilson 2009), but a new era of systems thinking promises to transform exposure science.

Exposure science is responding to advances in technology (Cohen Hubal 2009). Rapid improvements in computing hardware and software have led to the emergence of efficient computational approaches to collecting data, simulating complex processes, and systematically evaluating models. Simultaneously, diverse and “big” data are becoming increasingly available. New social media–based methods of obtaining perception and behavior information are being developed (Eysenbach 2009), and further development of low-cost sensors will soon empower “citizen scientists” to provide a broad range of data, including chemical concentrations, using omnipresent technologies such as smartphones (Dickinson et al. 2010; Snyder et al. 2013).

These public health drivers and novel scientific and technological advances are facilitating the development of computational exposure science as an emerging dimension of exposure science, akin to the emergence of computational toxicology more than a decade ago (el-Masri et al. 2002; Kavlock and Dix 2010). With various representations of computational exposure science beginning to appear in the literature (Dionisio et al. 2015; Isaacs et al. 2014; Shin et al. 2015; Wambaugh et al. 2013), this commentary is intended to define the emerging discipline, establish a conceptual framework, and provide some illustrative examples of research that is being conducted to advance the field of exposure assessment with regard to chemical ingredients of consumer products.

## Discussion

### Defining Computational Exposure Science

We define computational exposure science as the integration of advances in chemistry, computer science, mathematics, statistics, and social and behavioral sciences with new and efficient models and data collection methods to reliably and effectively forecast real-world exposures to natural and anthropogenic chemicals in the environment. Computational exposure science aims to link exposures to health outcomes through the application of environmental informatics and advanced computational tools, as previously envisioned (Cohen Hubal 2009; Cohen Hubal et al. 2010; Sheldon and Cohen Hubal 2009), and to take full advantage of scientific innovation and the resulting abundance of newly available information for predictive, rapid, and high-throughput exposure assessment.

Although computational exposure science is conceptualized in the spirit of computational biology, it builds on a rich history of computational models for understanding environmental science that dates to the early 1900s (NRC 2007a). For key environmental chemicals, decades of observational studies have enabled the evolution of empirical and mechanistic models that can reliably explain the distribution and fate of chemicals in biological and environmental media. (Tornero-Velez et al. 2012; Zartarian et al. 2012). Multimedia fate models have greatly expanded modeling capacity to large inventories of chemicals. These models predict human exposure using mechanistic mass balance equations and food web bioaccumulation calculations to describe transfer between environmental compartments (e.g., air, water, soil, biota) (Arnot et al. 2012; Bennett et al. 2002; Diamond et al. 1994) but require few inputs other than easily obtainable physical/chemical descriptors. In the era of big data and informatics, analytical solutions are giving way to more numerical, computational, and systems-focused methods. Nonetheless, a computational exposure science approach is not a replacement for traditional monitoring, survey, and modeling methods used in exposure science. Although computational exposure science provides the opportunity to examine a more expansive range of chemicals than can be investigated by the aforementioned traditional methods, it also carries much greater levels of uncertainty. Risk context (NRC 2009) should determine whether rapid or highly refined approaches are employed. Moreover, the two approaches complement each other: the screening-level results of predictive models can identify targets for measurement, and the acquisition of new data through measurements and field studies is needed to evaluate and improve computational exposure science methods (NRC 2007b).

### Applying Computational Exposure Science

**Figure 1:**
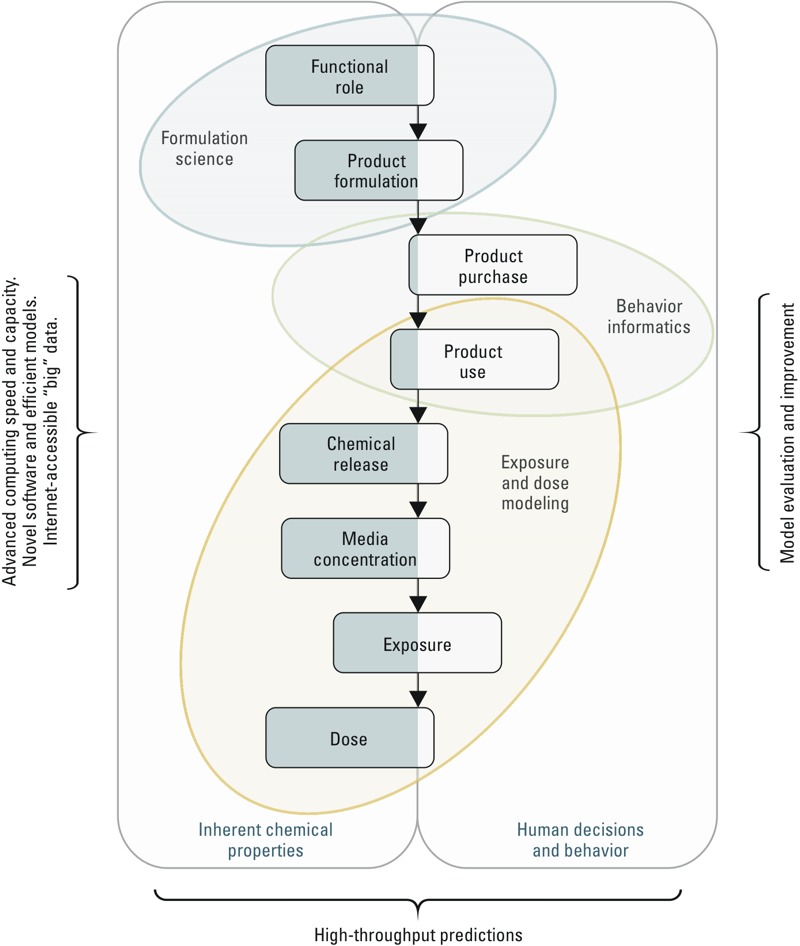
Framework for computational exposure science. The rounded rectangles represent components of computational exposure science required for high-throughput exposure assessment; their relative positions toward the left or the right represent the two generalized categories of source data (inherent chemical properties and human decisions, respectively).

Our ambitious goal for computational exposure science at the U.S. Environmental Protection Agency (EPA) is to rapidly and defensibly predict screening-level population exposure and intake dose rates for any existing or new chemical, even if few data exist beyond chemical structure. As depicted in Figure 1, understanding exposure to any chemical requires linkages from chemical functional role to formulated component of consumer products, to identification of scenarios involving chemical release, media concentrations, and human contact, and, ultimately, to models estimating uptake and dose. The functional role of a chemical (i.e., how it is used in processes or products) is determined by its inherent chemical properties, which are imparted by the chemical structure.

Predicting exposure and dose from chemical structure requires viable approaches for integrating information about the function of a chemical both with mechanistic fate and transport processes and with social and behavioral science descriptions of consumer product use and activities. Two distinct realms of information, or “source data,” that are critical for estimating exposure are illustrated in Figure 1. These realms represent *a*) inherent chemical, physical, and molecular properties; and *b*) decisions and other behaviors that govern product use. The individual components are arranged to visually indicate whether the data streams characterizing each component are mainly in the domain of “inherent chemical properties” or “human decisions and behavior” (beyond traditional exposure factors). For example, media concentrations can be predicted using a multimedia mass balance model and a chemical release factor, both of which can be parameterized using the inherent properties of the chemical (Arnot et al. 2012). Product purchase and use, however, are the result of complex individual-level decisions that drive overall consumer behaviors in a stochastic fashion (Rand and Rust 2011). The position of the “exposure” component reflects that the process depends as much on inherent properties as it does on complex social, psychological, and economic drivers.

The system of linkages depicted in Figure 1 may not seem like a radical departure from the traditional, linear source-to-dose continuum (Lioy 1990), nor is it intended as one. Instead, it expands on the modification proposed in the NRC report, *Exposure Science in the 21st Century* (Lioy and Smith 2013; NRC 2012). Specifically, this framework expands the “upstream” factors of exposure to formulation science (i.e., functional role, product formulation) and to behavior informatics (i.e., product purchase, product use), and it identifies the types of data streams required to parameterize each component. This addition of upstream processes provides a natural mechanism by which the product manufacturing stage of the product’s life cycle (i.e., product formulation) can be linked directly to human behavior.

The application of computational exposure science to characterize each process shown in Figure 1 for thousands of chemicals will require one or more of the following generalizable activities:

identification and acquisition of multiple data streams from traditional and nontraditional sources
extrapolation of model input parameters from data-rich to data-limited chemicals
integration of multiple data streams for large sets of chemicals through rapid, efficient, and reliable models
evaluation of model performance by systematic comparison of model predictions with measured values obtained through targeted or non-targeted analyses and by application of quantitative model sensitivity and uncertainty analyses to identify key data limitations and sources of uncertainty
continuous acquisition and incorporation of new data streams that address key uncertainties and performance of refinements in an iterative and self-consistent manner.

An immediate challenge in computational exposure science is identifying and integrating data streams, particularly those outside of the traditional realm of exposure science, that are essential for understanding, parameterizing, and evaluating interactions between (chemical) stressors and (human) receptors. For example, commercial market research data and internet search volume analytics remain largely unexploited for understanding consumer behaviors and their differences by region and demographics. Novel analytical tools (e.g., social network analysis, natural language processing) must be explored to facilitate the integration of nontraditional data streams into exposure assessment, just as ontologies are being developed to integrate exposure information with other disciplines (Goldsmith et al. 2012; Mattingly et al. 2012).

To parameterize the processes illustrated in Figure 1 for the vast number of data-limited chemicals in commerce, it is necessary to extrapolate chemical–behavior patterns (fate, transport, intake, etc.) from those of relatively data-rich chemicals [often using quantitative structure–activity relationship (QSAR) methodologies], with the explicit understanding that such extrapolation is fraught with uncertainty and demands empirical evaluation (Molitor et al. 2007; Oreskes 1998; Wambaugh et al. 2013).

Innovative modeling approaches are needed to understand relationships among data sources of varying complexity and quality and exposure-related factors, processes, and monitoring data. These approaches include machine-learning classification models, which have already proved to be well-suited for pharmacokinetic and hazard-related contexts (Freitas et al. 2015; Liu et al. 2015; Zang et al. 2013), and agent-based models, which provide a new opportunity to predict exposure-relevant behavior as a function of characteristics of individuals, their environments, and their interactions (Luke and Stamatakis 2012).

A clear understanding of domain of applicability (i.e., the set of conditions under which use of the model is scientifically defensible) is critical for the reliable application of models, as is appropriately quantifying the precision of mathematical models, evaluating their predictive value, and characterizing associated uncertainties. Care must be taken to ensure that the models truly reflect their assumed underlying theoretical constructs, particularly when relying on big data (Lazer et al. 2014; Oreskes 1998). Conventional evaluations of model predictions against available personal measurement data, along with advances in the computational implementation of statistical methods for model and data evaluation (Markov chain Monte Carlo sampling for Bayesian inference, in particular), provide a path forward for such evaluation (Molitor et al. 2007; Wambaugh et al. 2013; Zartarian et al. 2012). The results of such evaluation will guide the acquisition and incorporation of additional data to address key uncertainties and to further refine models (NRC 2007a).

### Current Research Activities and Examples

The application of computational exposure science as described above has led to a set of strategic research efforts by the U.S. EPA to advance high-throughput exposure predictions. Below, we provide examples describing the development and application of methods for assessing exposure to consumer product chemicals pertaining to *a*) the development of reliable, computationally efficient predictive models; *b*) the identification, acquisition, and analysis of data supporting high-throughput exposure model parameterization and model evaluation; and *c*) the extrapolation of available data to predict behaviors of large inventories of data-limited chemicals.

For proof of concept, our current focus has been on assessing exposures to chemical ingredients of consumer products under the construct that exposure to a chemical is a function of the type of product in which the chemical can be found and human activity patterns related to that product. The general strategy has been to identify products, map products to chemical ingredients, map products to use patterns and exposure scenarios, and then employ scenarios to model chemical exposures by route and pathway.

To supplement the National Library of Medicine’s (NLM’s) Household Products Database (NLM 2015) for information on product ingredients, the U.S. EPA has built the Consumer Product Chemical Profiles database (CPCPdb) (Goldsmith et al. 2014). Using optical character recognition and automated parsing to extract information from publicly available product material safety data sheets, CPCPdb has been populated with roughly 1,800 unique chemicals in 353 product categories. The U.S. EPA has also developed a database [Chemical Product Categorization database (CPCat)] of various levels of chemical use information for more than 40,000 chemicals (Dionisio et al. 2015). CPCPdb has been consolidated into CPCat, and both databases are available through the U.S. EPA’s online warehouse of chemical data, known as the Aggregated Computational Toxicology Resource (ACToR) (U.S. EPA 2015). Use-related data within ACToR have already been shown to correlate with exposures inferred from biomonitoring (Wambaugh et al. 2014), and these databases provide a foundation for the development of modeling systems to predict chemical functional use (based on properties), and then, from functional use, the types of products in which chemicals are likely to be found (“use profiles”).

As QSARs are used to extrapolate physicochemical and pharmacokinetic properties across chemicals, similar models are being developed to determine relationships between predicted properties or structural descriptors and chemical functional role in products and to probabilistically predict weight fractions of consumer product ingredients based on the functions they perform in products. Such analyses may eventually aid in identification of the underlying inherent chemical properties (molecule features) that confer the desired properties. As such, these computational exposure modeling methods can be repurposed to design safer ingredients or to identify safer, readily available alternatives.

For the purposes of high-throughput exposure assessment, the U.S. EPA has developed a new modeling approach, the Stochastic Human Exposure and Dose Simulation–High-Throughput (SHEDS-HT) model (Isaacs et al. 2014), that combines use profiles with consumption information and then maps these factors to exposure scenarios. SHEDS-HT is based on the methods and algorithms of the SHEDS model for multimedia pollutants, commonly known as SHEDS Multimedia (Zartarian et al. 2012), but the fugacity, residential, and dietary modules have been numerically and operationally reduced to decrease user burden and to increase run speed while maintaining critical features. A fugacity-based source-to-concentration module estimates indoor concentrations by media (air, dust, and surfaces). Concentration estimates, along with relevant exposure factors and human activity data, are then used by the model to rapidly generate population-specific distributions of potential residential exposures via dermal, nondietary ingestion, and inhalation pathways. Estimated population dietary exposures are combined with the residential exposure predictions to produce total exposure estimates.

**Figure 2:**
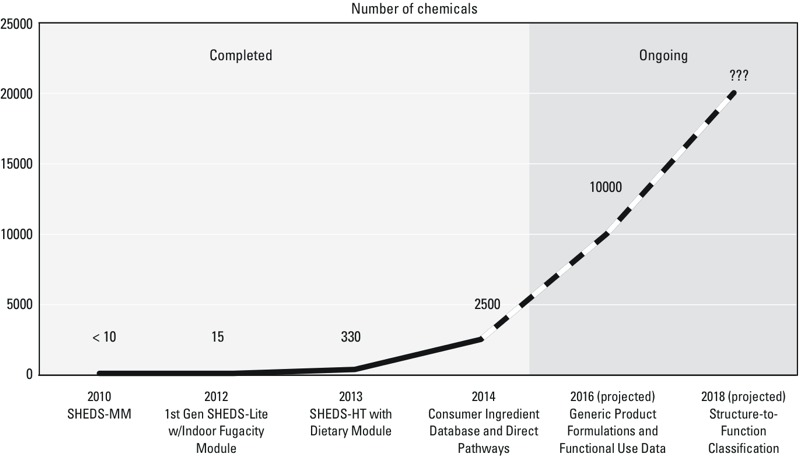
The number of chemicals for which probabilistic exposure assessment has been performed has increased dramatically over the past 2 years. Current efforts toward the development of generic product formations and structure-to-function property–activity relationships will produce accelerated rates of evaluation.

The development of SHEDS-HT, together with informatics-based methods of obtaining chemical use information, led to a significant increase in the speed of probabilistic exposure assessment and in the number of chemicals assessed. For example, the premier, higher-tier SHEDS Multimedia (SHEDS-MM) model had been applied to fewer than 10 chemicals over the past 15 years in support of high-priority regulatory support activities (Figure 2). In contrast, the first generation of SHEDS-HT extended the number to 15 by using less-detailed inputs and appropriate measurement surrogates. The addition of the simplified dietary module 1 year later increased the number of chemicals that had been investigated to 330, and subsequent enhancement with information from consumer product ingredient databases brought the number of chemicals assessed to 2,500 (Isaacs et al. 2014). We anticipate that current efforts aimed at the development of structure-to-function relationships will produce an accelerated rate of model parameterization that will enable screening-level forecasts for ≥ 10,000 chemicals by 2016 and perhaps twice that number within the subsequent 2 years.

To assess new models such as SHEDS-HT and foster confidence for their application in regulatory settings, we are developing techniques to evaluate model predictions that are both systematic (e.g., to measure performance across a broad range of chemical stressors) and empirical (e.g., to determine how reliably the available data support model estimates). We have relied on a well-defined framework using a Bayesian statistical methodology to draw inferences from biomonitoring data for evaluating model predictions. This framework, called Systematic Empirical Evaluation of Models (Wambaugh et al. 2013, 2014), provides consensus exposure forecasts from multiple models along with an empirical determination of uncertainty in the resulting model predictions. This framework is a direct example of how traditional biomonitoring and exposure data can be used in concert with (perhaps less certain) computational exposure science modeling results.

## Conclusions

The emerging discipline of computational exposure science represents an evolution of exposure science toward the identification of new data sources and the application of innovative modeling techniques for understanding and quantifying human exposures to chemicals. The success of computational exposure science as a discipline will require that we also design and implement new research to collect the critical monitoring data needed to evaluate and improve the reliability of the next generation of models and to reduce the uncertainty in chemical exposure model predictions for screening and prioritization purposes and other applications (e.g., ecological impact analysis, life cycle analysis, broad sustainability evaluations). Performing nontargeted analysis of the chemicals present in biological and environmental media using high-resolution mass spectrometry platforms will play a key role in developing these models. The wealth of data produced by nontargeted measurement techniques can be used to generate and test hypotheses regarding the fate of chemicals as a function of, for example, their physical–chemical properties, use (applicative or functional), and source distance [near-field (applied to the body or released indoors) vs. far-field (released to the outdoor environment)], but innovative data analysis methods beyond those described herein will be required. Results from both nontargeted analysis and computational models will be used to optimize future exposure data collection efforts. The symbiotic relationship between methods, measurements, and modeling traditionally realized in exposure science is no less relevant within computational exposure science, but here, this relationship takes on a systems-focused and high-throughput form.

Although the examples provided herein focus on U.S. EPA research, it should be acknowledged that other groups are also engaged in advancing computational exposure science. For example, the National Institute of Environmental Health Sciences–funded Health and Exposome Research Center: Understanding Lifetime Exposures (HERCULES) (HERCULES Exposome Research Center 2015) and the European Union–funded Health and Environment-wide Associations based on Large population Surveys (HEALS) (HEALS Consortium 2015) projects are both bringing together novel technologies, data analysis techniques, and modeling tools to support exposome studies. Whereas our examples emphasize human exposure to consumer-product chemicals, computational exposure science methods are also amenable for broader application, and similar approaches are already being evaluated for ecological receptors. As the technology rapidly evolves, the potential applications of these methods will expand, and the promise of minimizing significant adverse impacts of chemicals on human health will become more attainable.

A critical mass of research around the themes of exposure modeling, statistics, and novel data streams is affirming computational exposure science as sufficiently distinct and mature to warrant description within the scientific literature. The emergence of computational exposure science has been motivated by both need and opportunity, in parallel with the earlier emergence of computational toxicology from toxicology. The availability of toxicity testing data for thousands of chemicals highlights the need for an exposure context to gauge risk and inform regulation (NRC 2007b; Thomas et al. 2013; Tice et al. 2013) as well as the need for exposure assessment to not be the rate-limiting step for high-throughput risk assessment. By identifying and defining this new and rapidly emerging dimension of exposure science, we hope to foster its continued development in support of protection of health and the environment.
